# Effect of dihydropyrimidine dehydrogenase single nucleotide polymorphisms on prognosis of breast cancer patients with chemotherapy

**DOI:** 10.18632/oncotarget.23033

**Published:** 2017-12-08

**Authors:** Fengxia Qin, Huikun Zhang, Yong Huang, Limin Yang, Feng Yu, Xiaoli Liu, Li Fu, Feng Gu, Yongjie Ma

**Affiliations:** ^1^ Department of Breast Cancer Pathology and Research Laboratory, Tianjin Medical University Cancer Institute and Hospital, Tianjin, China; ^2^ Department of Tumor Cell Biology, Tianjin Medical University Cancer Institute and Hospital, Tianjin, China; ^3^ National Clinical Research Center for Cancer, Tianjin, China; ^4^ Tianjin’s Clinical Research Center for Cancer, Tianjin, China; ^5^ Key Laboratory of Cancer Prevention and Therapy, Tianjin, China; ^6^ Key Laboratory of Breast Cancer Prevention and Therapy, Tianjin Medical University, Ministry of Education, Tianjin, China

**Keywords:** DPYD, fluoropyrimidine, breast cancer, prognosis, chemotherapy

## Abstract

Defining biomarkers that predict therapeutic effects and adverse events is a crucial mandate to guide patient selection for personalized cancer treatments. DPD (dihydropyrimidine dehydrogenase, encoded by *DPYD* gene) is the initial and rate-limiting enzyme of metabolic pathway of fluoropyrimidines, and fluoropyrimidines are common used drug therapies for breast cancer. Previous studies on *DPYD* polymorphism were mainly focused on its association with fluoropyrimidines toxicity. In our present study, 5 *DPYD* single nucleotide polymorphisms status was detected from tumor tissues of 331 invasive breast cancer patients using standard techniques. We for the first time investigated the prognostic significance of *DPYD* polymorphisms in breast cancer. We demonstrated non-luminal breast cancer patients carrying *DPYD* c.1627A>G AG/GG treated with fluoropyrimidine-based regimen presented a shorter overall survival and progression-free survival than carriers treated with non-fluoropyrimidine regimen. However, non-luminal *DPYD* c.1627A>G AG/GG carriers treated with TE (taxane and anthracycline)-based regimen showed a better prognosis compared with carriers treated with non-TE regimen. Our results suggested TE-based chemotherapy was a suitable regimen for non-luminal patients with *DPYD* c.1627A>G AG/GG genotype and fluoropyrimidine-based regimen should not be recommended for those patients. Our findings provided a novel strategy, which will guide clinicians to choose more precise chemotherapy treatment for breast cancer patients.

## INTRODUCTION

Precision medicine in oncology is the result of an increasing awareness of patient specific clinical features. It does not only mean utilization of tumor biomarkers, but also relevant germline-mutation-detections in drug metabolizing enzymes and transporters, which have been shown to impact drug response, providing rationale for individualized treatment [1].

Fluoropyrimidines are the most prevalent and effective chemotherapeutic agents used for the systemic treatment of various malignancies including gastrointestinal, breast, pancreas and head and neck cancers [2–5]. DPD (dihydropyrimidine dehydrogenase), encoded by *DPYD* gene, is the initial and rate-limiting enzyme of the metabolic pathway of fluoropyrimidines, such as 5-Fu, capecitabine and tegafur [6–8]. The clinical importance of DPD was initially identified due to severe or lethal toxicity in patients given fluoropyrimidines who are deficient in or have low levels of DPD activity [9–11]. Since then, more than 50 *DPYD* polymorphisms have been reported to cause fluoropyrimidine-associated toxicity in the treatment of malignancies such as colorectal carcinoma, gastroesophageal cancer and lymphoblastic leukemia [12–14]. Recently, emerging evidence indicated that *DPYD* polymorphisms could contribute to tumorgenesis and influence the chemosensitivity as well as clinical outcomes of cancer patients. It was reported that *DPYD* single nucleotide polymorphisms (SNPs) led to an increased risk of ovarian cancer and gastrointestinal tumors patients with *DPYD* c.1627A>G AG/GG genotype presented low chemosensitivity to fluorouracil-based adjuvant treatment [15, 16]. Furthermore, *DPYD* SNPs (rs1760217) were significantly associated with reduced survival in pancreatic cancer patients [17]. However, the prognostic significance of *DPYD* polymorphisms in breast cancer has rarely been investigated.

In our present study, 5 *DPYD* SNPs status (c.74A>G, c.85T>C, c.1627A>G, c.1896T>C, c.2194G>A) were detected in tumor tissues from 331 invasive breast cancer patients. We demonstrated for the first time that *DPYD* SNPs status was associated with breast cancer prognosis, especially the impact of c.1627A>G polymorphism on prognosis of non-luminal subtype. We found that non-luminal breast cancer patients carrying *DPYD* c.1627A>G AG/GG genotype treated with fluoropyrimidine-based regimen presented a shorter overall survival (OS) and progression-free survival (PFS) compared with carriers treated with non-fluoropyrimidine regimen. However, non-luminal *DPYD* c.1627A>G AG/GG genotype carriers treated with TE (taxane and anthracycline)-based regimen showed a better prognosis compared with carriers treated with non-TE regimen. All these results suggested that TE-based chemotherapy was a suitable regimen for non-luminal breast cancer patients with *DPYD* c.1627A>G AG/GG genotype and fluoropyrimidine-based chemotherapy should not be recommended for these patients. Our findings provided a novel strategy, which will guide clinicians to choose more precise chemotherapy treatment for breast cancer patients.

## RESULTS

### Association between *DPYD* SNPs status and prognosis of patients with fluoropyrimidine-based chemotherapy, especially in non-luminal subtype breast cancer

Primers for 5 *DPYD* SNPs amplifications were presented in Table 1 and genotypic frequencies and characteristics of 331 breast cancer specimens were shown in Table 2. In this study, c.74A>G and c.2194G>A SNPs were excluded due to a limited frequency (minor allele frequency**<**5%). The observed genotype frequencies of c.85T>C, c.1627A>G and c.1896T>C were all in Hardy-Weinberg equilibrium and they were analyzed in the following studies. Example sequence traces of *DPYD* SNPs were shown in Supplementary Figure 1.

**Table 1:** PCR primer sequences

PCR reaction	Primer sequences
**c.74A>G and c.85T>C**	
** forward**	**5’-GCAGTGAACTGAGATTGTACCACT-3’**
** reverse**	**5’-CTTGCCTTACAATGTGTGGAG-3’**
**c.1627A>G**	
** forward**	**5’-TATTATATGGACAATTTAGAT-3’**
** reverse**	**5’-GATAGACATTTCTATATGACT-3’**
**c.1896T>C**	
** forward**	**5’-TCATCAGGACATTGTGACAAAT-3’**
** reverse**	**5’-CTTTCTATGCATCAGCAAAGC-3’**
**c.2194G>A**	
** forward**	**5’-GTAGGAGTTAAATTAGTGAAG-3’**
** reverse**	**5’-AGCAACCTCCAAGAAAGCACA-3’**

**Table 2:** *DPYD* SNPs information and genotypic frequencies

Genotype	Location	Effect	Cases (%)	HWE *P* value^a^
**c.85T>C**	**Exon 2**	**Cys29Arg**		**0.201**
** TT**			**273 (82.5)**	
** TC**			**53 (16.0)**	
** CC**			**5 (1.5)**	
**c.1627A>G**	**Exon 13**	**Ile543Val**		**0.144**
** AA**			**193 (58.3)**	
** AG**			**113 (34.1)**	
** GG**			**25 (7.6)**	
**c.1896T>C**	**Exon 14**	**Phe632Phe**		**0.161**
** TT**			**256 (77.3)**	
** TC**			**67 (20.3)**	
** CC**			**8 (2.4)**	
**c.2194G>A**	**Exon 18**	**Val732Ile**		**0.824**
** GG**			**323 (97.6)**	
** GA**			**8 (2.4)**	
** AA**			**0 (0)**	
**c.74A>G**	**Exon 2**	**His25Arg**		**<0.001**
** AA**			**328 (99.1)**	
** GA**			**0 (0)**	
** GG**			**3 (1.6)**	

**HWE: Hardy-Weinberg equilibrium.**

**^a^Two-sided χ^2^ test.**

We found that non-wild type *DPYD* (patients with c.85T>C TT, c.1627A>G AA and c.1896T>C TT simultaneously were identified as wild type *DPYD* group, whereas patients with whichever of mutations at c.85T>C, c.1627A>G or c.1896T>C were identified as non-wild type *DPYD* group) was not correlated with clinicopathological characteristics (Supplementary Table 1).

**Figure 1:**
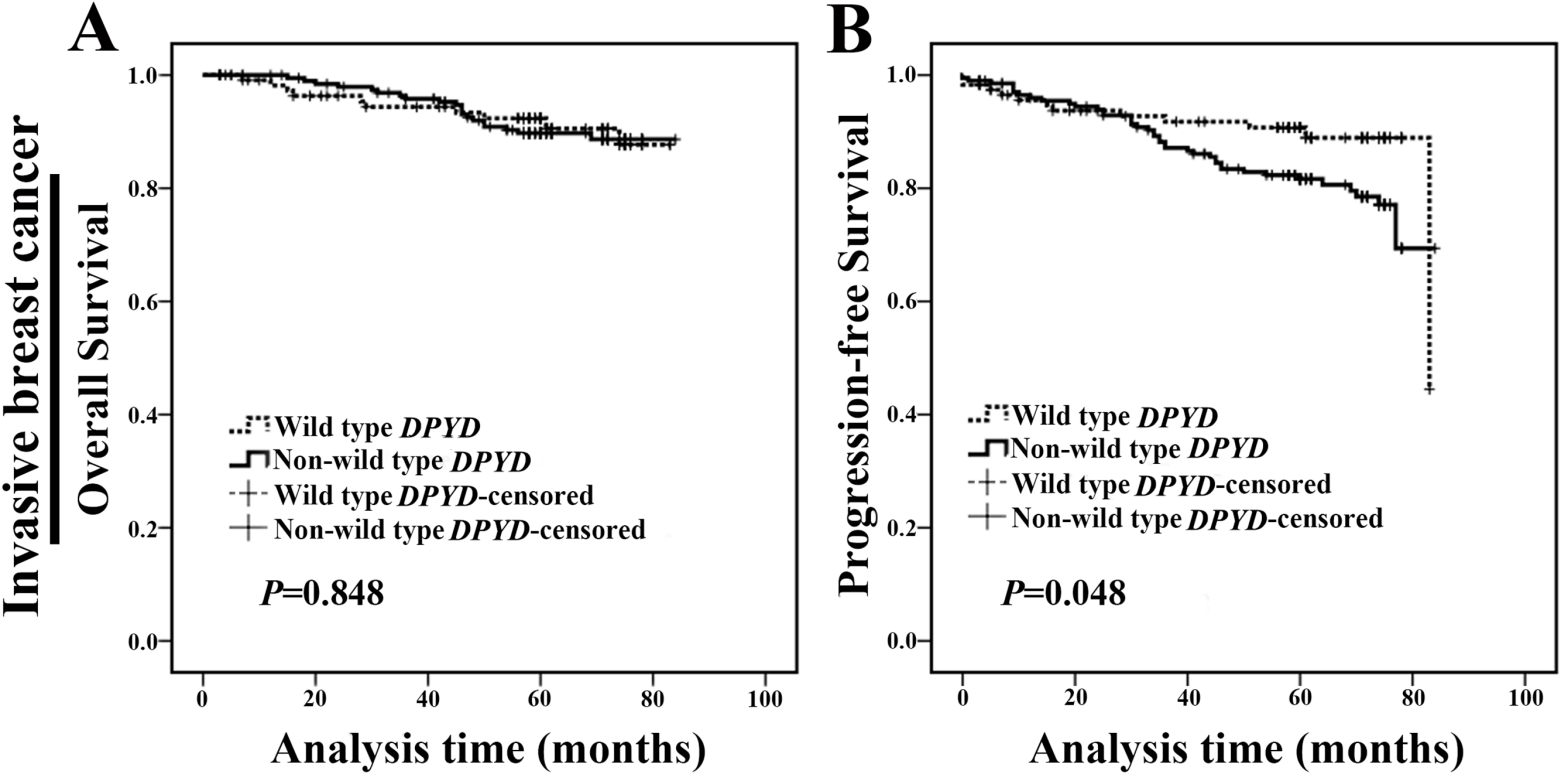
Relationship between *DPYD* SNPs status and breast cancer patients prognosis **(A)** Patients with non-wild type *DPYD* exhibited a similar overall survival (OS) compared with wild type *DPYD* carriers (log-rank test). **(B)** Patients with non-wild type *DPYD* exhibited a shorter progression-free survival (PFS) compared with wild type *DPYD* carriers.

**Figure 2:**
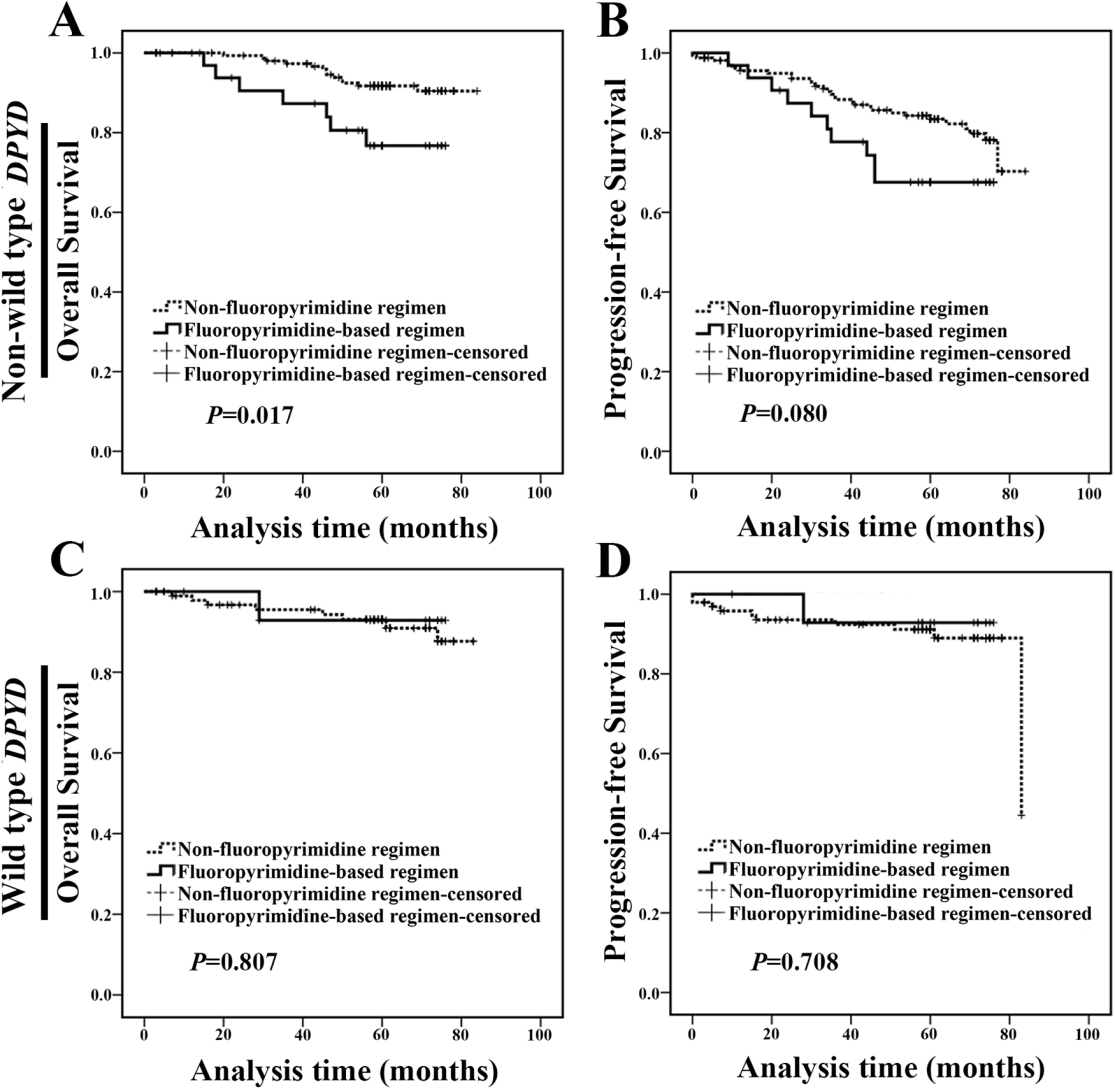
Non-wild type *DPYD* carriers treated with fluoropyrimidine-based regimen exhibited a poor prognosis **(A-B)** Non-wild type *DPYD* carriers treated with fluoropyrimidine-based regimen exhibited a shorter OS compared with those treated with non-fluoropyrimidine regimen. **(C-D)** Wild type *DPYD* carriers treated with fluoropyrimidine-based regimen exhibited a similar OS and PFS compared with those with non-fluoropyrimidine regimen.

To investigate the influence of *DPYD* SNPs on breast cancer prognosis, we compared the clinical outcome of patients with wild type or non-wild type *DPYD* and no obvious difference was found between them in OS analysis (*P*=0.848, Figure 1). However, non-wild type *DPYD* carriers treated with fluoropyrimidine-based regimen exhibited a shorter OS compared with carriers treated with non-fluoropyrimidine regimen (*P*=0.017, Figure 2A and 2B). We did not find any statistical difference in clinicopathologic characteristics between the two populations at baseline (Supplementary Table 2). Meanwhile, for wild type *DPYD* carriers, the clinical outcome of patients treated with fluoropyrimidine-based regimen was similar to carriers treated with non-fluoropyrimidine regimen (Figure 2C and 2D). It suggested that SNPs status of *DPYD* was associated with effect of fluoropyrimidine-based treatment.

**Figure 3:**
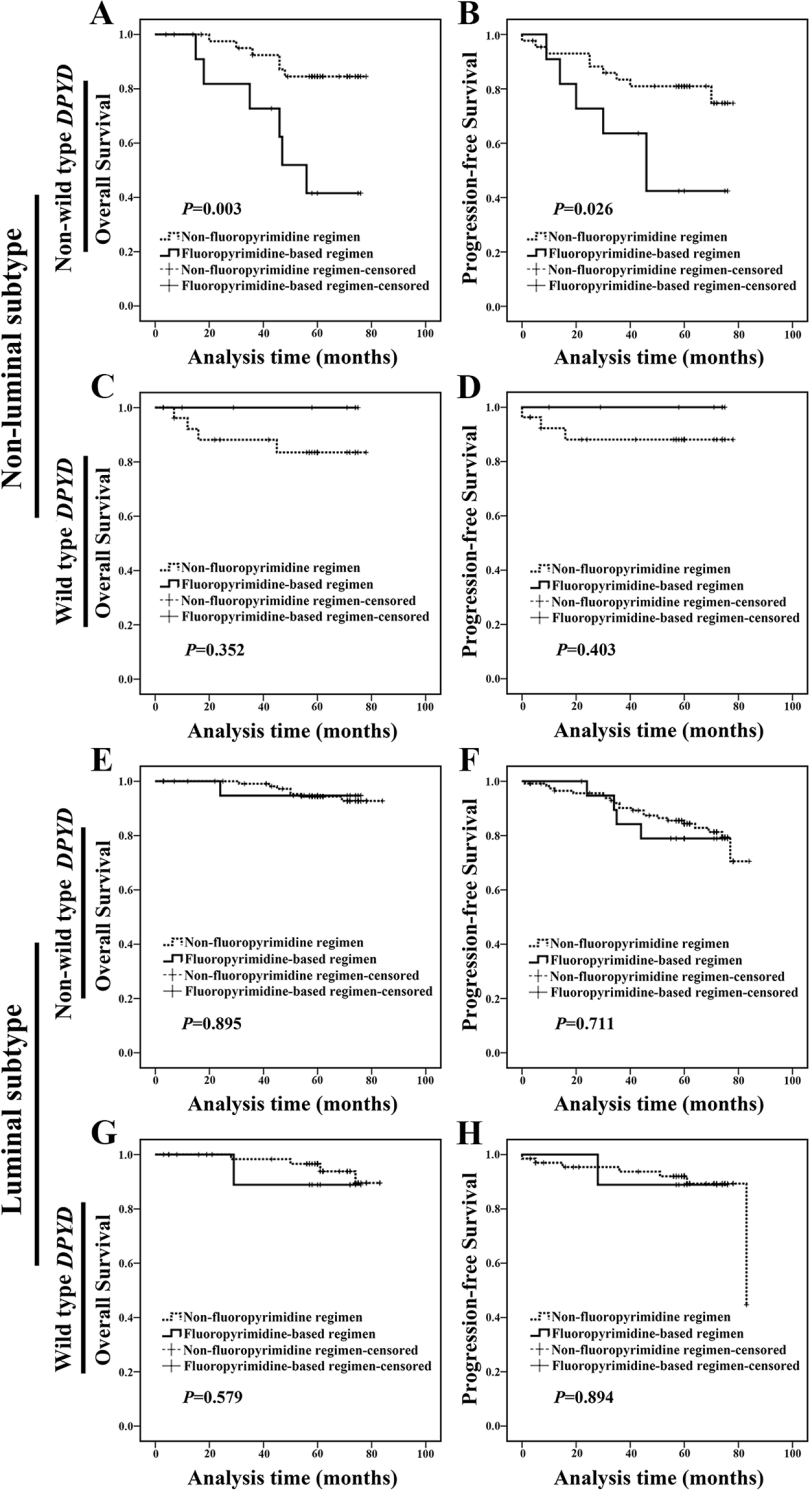
Non-wild type *DPYD* indicated a poor prognosis in non-luminal breast cancer patients treated with fluoropyrimidine-based regimen **(A-B)** For non-luminal subtype, non-wild type *DPYD* carriers treated with fluoropyrimidine-based regimen exhibited a shorter OS and PFS compared with carriers treated with non-fluoropyrimidine regimen. **(C-D)** For non-luminal subtype, wild type *DPYD* carriers treated with fluoropyrimidine-based regimen exhibited a similar OS and PFS compared with those treated with non-fluoropyrimidine regimen. **(E-F)** For luminal subtype, non-wild type *DPYD* carriers treated with fluoropyrimidine-based regimen exhibited a similar OS and PFS compared with those treated with non-fluoropyrimidine regimen. **(G-H)** For luminal subtype, wild type *DPYD* carriers treated with fluoropyrimidine-based regimen exhibited a similar OS and PFS compared with those treated with non-fluoropyrimidine regimen.

Subsequently, the clinical significance of *DPYD* SNPs in different breast cancer molecular subtypes was examined. In non-luminal subgroup, non-wild type *DPYD* carriers treated with fluoropyrimidine-based regimen exhibited a worse prognosis compared with carriers treated with non-fluoropyrimidine regimen (Figure 3A-3D). We did not find any statistical difference in clinicopathologic characteristics between the two populations at baseline (Supplementary Table 3). However, in luminal subgroup, patients treated with fluoropyrimidine-based regimen exhibited a similar outcome compared with those treated with non-fluoropyrimidine regimen, regardless of their *DPYD* SNPs status (wild type or non-wild type) (Figure 3E-3H). It suggested that non-luminal patients with non-wild type *DPYD* were unable to benefit from fluoropyrimidine-based chemotherapy.

### Association between c.1627A>G AG/GG and prognosis of patients with fluoropyrimidine-based chemotherapy, especially in non-luminal subtype breast cancer

**Figure 4:**
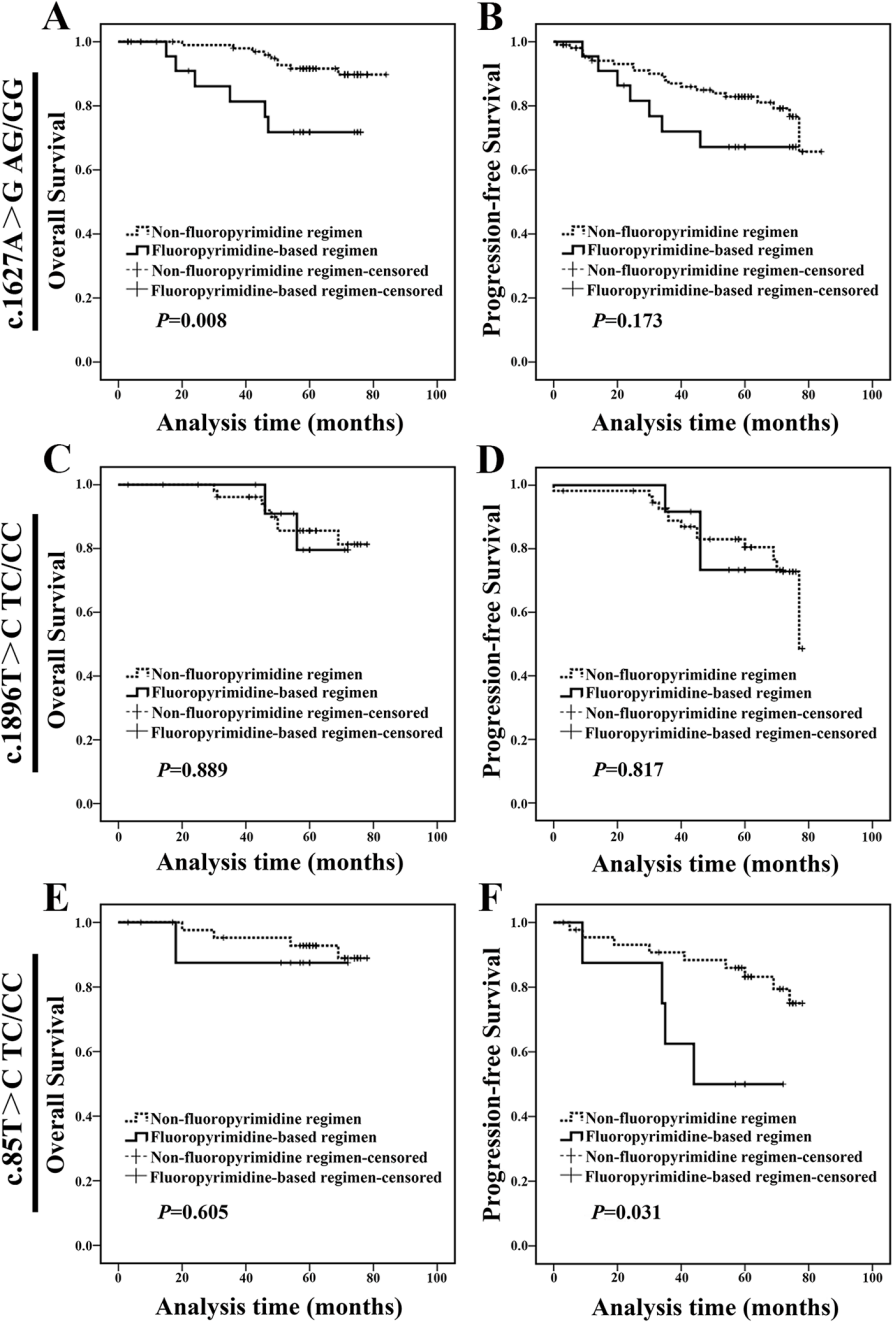
c.1627A>G AG/GG genotype carriers treated with fluoropyrimidine-based regimen exhibited a worse prognosis **(A-B)** c.1627A>G AG/GG genotype carriers treated with fluoropyrimidine-based regimen exhibited a shorter OS than those treated with non-fluoropyrimidine regimen. **(C-D)** c.1896T>C TC/CC genotype carriers treated with fluoropyrimidine-based regimen exhibited a similar OS and PFS compared with those treated with non-fluoropyrimidine regimen. **(E-F)** c.85T>C TC/CC genotype carriers treated with fluoropyrimidine-based regimen exhibited a similar OS compared with those treated with non-fluoropyrimidine regimen.

In the following studies, we started to focus on the clinical significance of 3 polymorphisms, individually. For c.1627A>G AG/GG genotype carriers, patients treated with fluoropyrimidine-based regimen exhibited a shorter OS and a tendency of shorter PFS compared with those treated with non-fluoropyrimidine regimen (Figure 4A and 4B). We did not find any difference in clinicopathologic characteristics between two populations at baseline (Supplementary Table 4). However, for c.1896T>C TC/CC genotype carriers, patients treated with fluoropyrimidine-based regimen exhibited a similar OS and PFS compared with those treated with non-fluoropyrimidine regimen (Figure 4C and 4D). For c.85T>C TC/CC genotype carriers, patients treated with fluoropyrimidine-based regimen exhibited a similar OS and a shorter PFS compared with those treated with non-fluoropyrimidine regimen (Figure 4E and 4F). These results indicated that patients with c.1627A>G AG/GG genotype could not benefit from fluoropyrimidine-based regimen.

**Figure 5:**
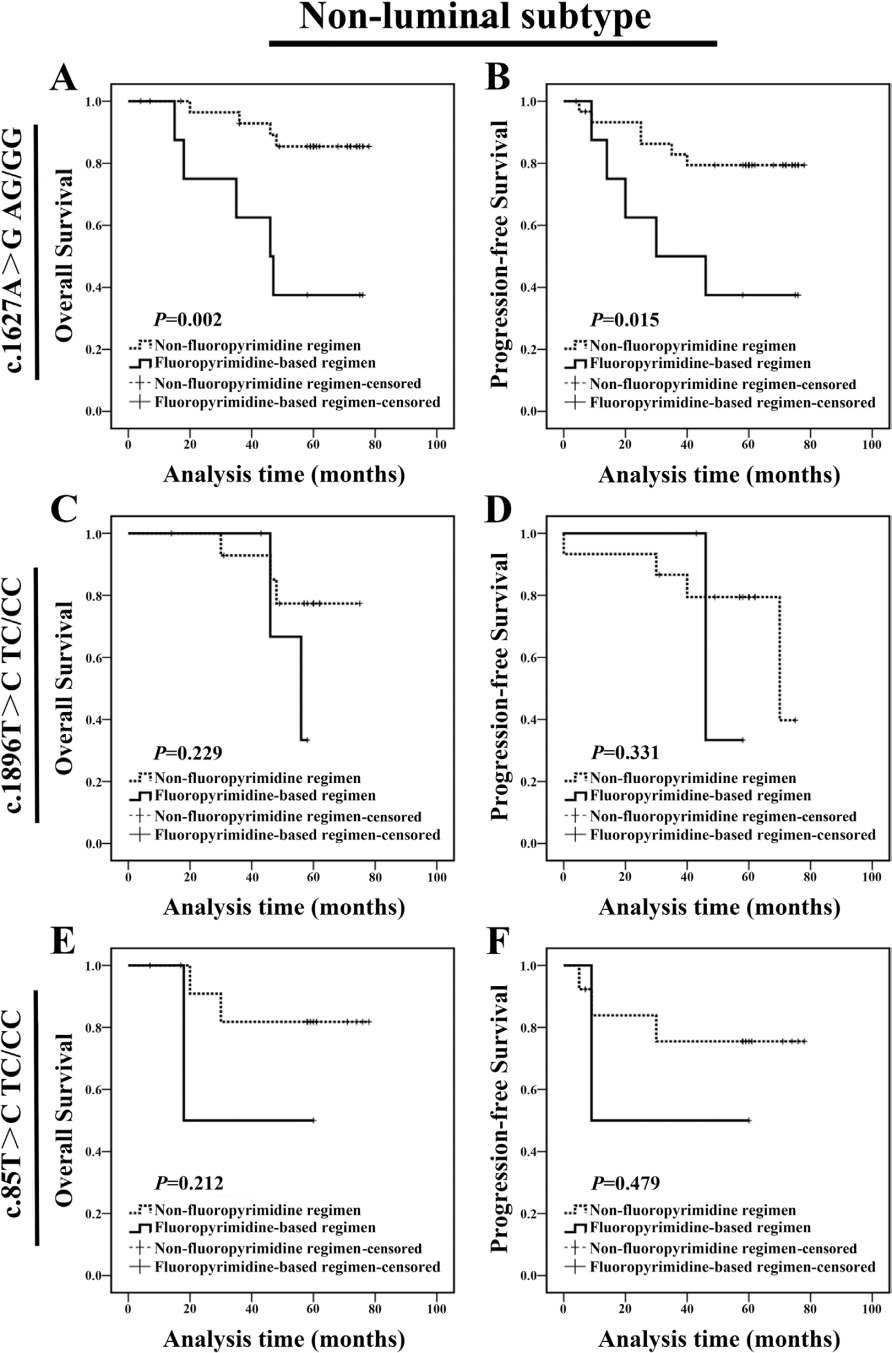
Non-luminal c.1627A>G AG/GG genotype carriers treated with fluoropyrimidine-based regimen exhibited a poor prognosis **(A-B)** For non-luminal subtype, c.1627A>G AG/GG genotype carriers treated with fluoropyrimidine-based regimen exhibited a shorter OS and PFS than those treated with non-fluoropyrimidine regimen. **(C-D)** For non-luminal subtype, c.1896T>C TC/CC genotype carriers treated with fluoropyrimidine-based regimen exhibited a similar OS and PFS compared with those treated with non-fluoropyrimidine regimen. **(E-F)** For non-luminal subtype, c.85T>C TC/CC genotype carriers treated with fluoropyrimidine-based regimen exhibited a similar OS and PFS compared with those with non-fluoropyrimidine regimen.

Next, in-depth analysis was performed to identify which molecular subtype patients with c.1627A>G AG/GG genotype could not benefit from fluoropyrimidine-based regimen. For non-luminal subtype, c.1627A>G AG/GG genotype carriers treated with fluoropyrimidine-based regimen showed a shorter OS and PFS compared with carriers treated with non-fluoropyrimidine regimen (Figure 5A and 5B). We did not find any difference in clinicopathologic characteristics between two populations at baseline (Supplementary Table 5). Meanwhile, c.1896T>C TC/CC genotype carriers treated with fluoropyrimidine-based regimen exhibited a similar OS and PFS (Figure 5C and 5D) compared with those treated with non-fluoropyrimidine regimen; and c.85T>C TC/CC genotype patients treated with fluoropyrimidine-based regimen exhibited a similar prognosis compared with carriers treated with non-fluoropyrimidine regimen (Figure 5E and 5F). These results indicated that non-luminal subtype patients with c.1627A>G AG/GG could not benefit from fluoropyrimidine-based chemotherapy. Notably, luminal c.1627A>G AG/GG carrier treated with fluoropyrimidine-based regimen exhibited a similar prognosis compared with carriers treated with non-fluoropyrimidine regimen (OS: *P*=0.888, PFS: *P*=0.718; Supplementary Figure 2).

### Association between c.1627A>G AG/GG and prognosis of patients with TE-based chemotherapy, especially in non-luminal subtype breast cancer

**Figure 6:**
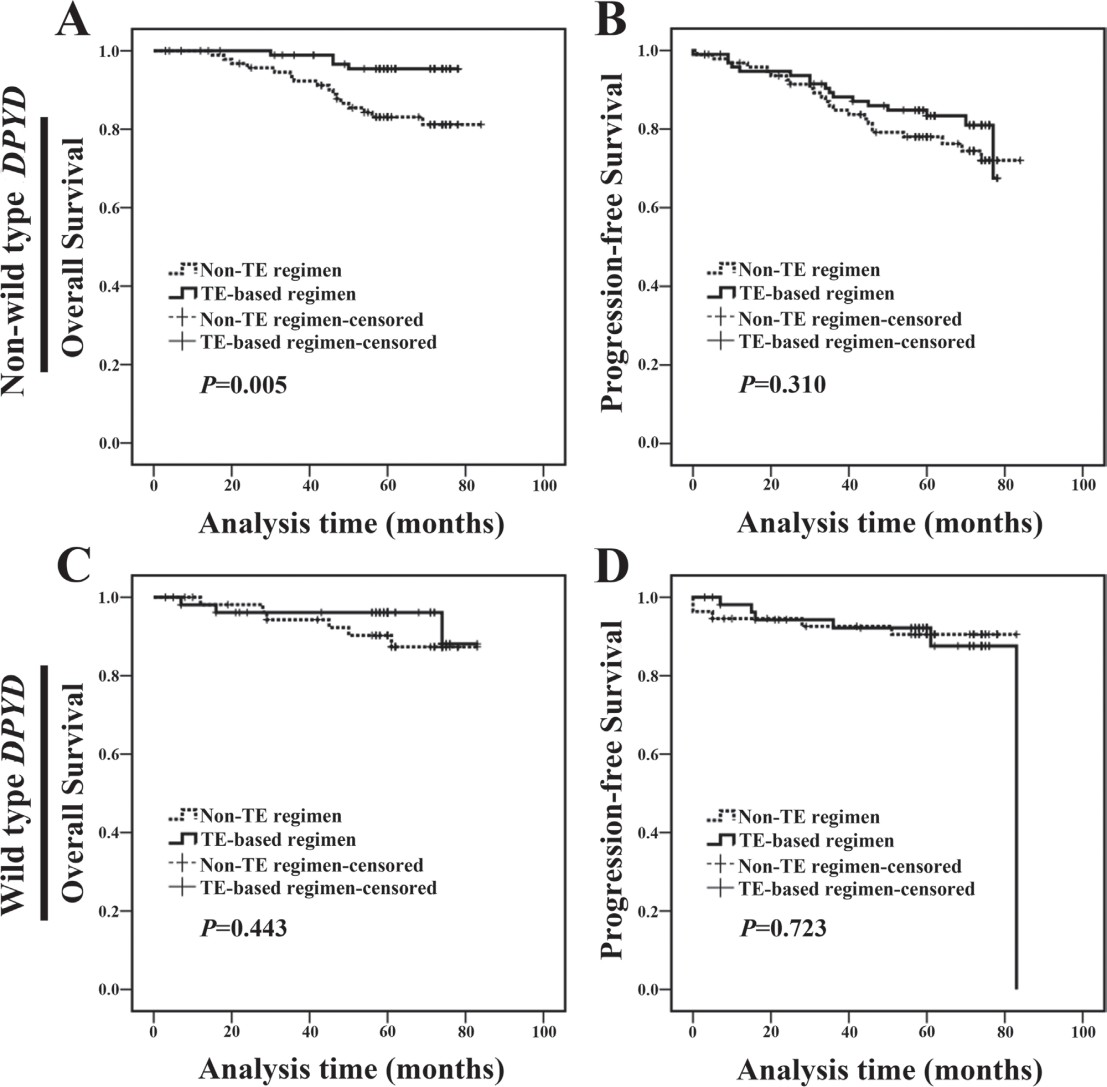
Non-wild type *DPYD* indicated a better prognosis in breast cancer patients treated with TE-based regimen **(A-B)** Non-wild type *DPYD* carriers treated with TE-based regimen exhibited a longer OS compared with those treated with non-TE regimen. **(C-D)** Wild type *DPYD* carriers treated with TE-based regimen exhibited a similar OS and PFS compared with those treated with non-TE regimen.

Besides fluoropyrimidine-based chemotherapy, TE (taxane and anthracycline) based therapies have been applied widely as the first-line treatment of breast cancer [18]. In the following experiments, we examined the relationship between *DPYD* SNPs and the effect of TE-based therapies. We found that non-wild type *DPYD* carriers treated with TE-based regimen exhibited a longer OS compared with carriers treated with non-TE regimen (Figure 6A and 6B). We did not find any statistical difference in clinicopathologic characteristics between the two populations at baseline (Supplementary Table 6). Meanwhile, for wild type *DPYD* carriers, the clinical outcome of patients treated with TE-based regimen was similar to those treated with non-TE regimen (Figure 6C and 6D). These results suggested that breast cancer patients with non-wild type *DPYD* were more sensitive to TE-based chemotherapy.

**Figure 7:**
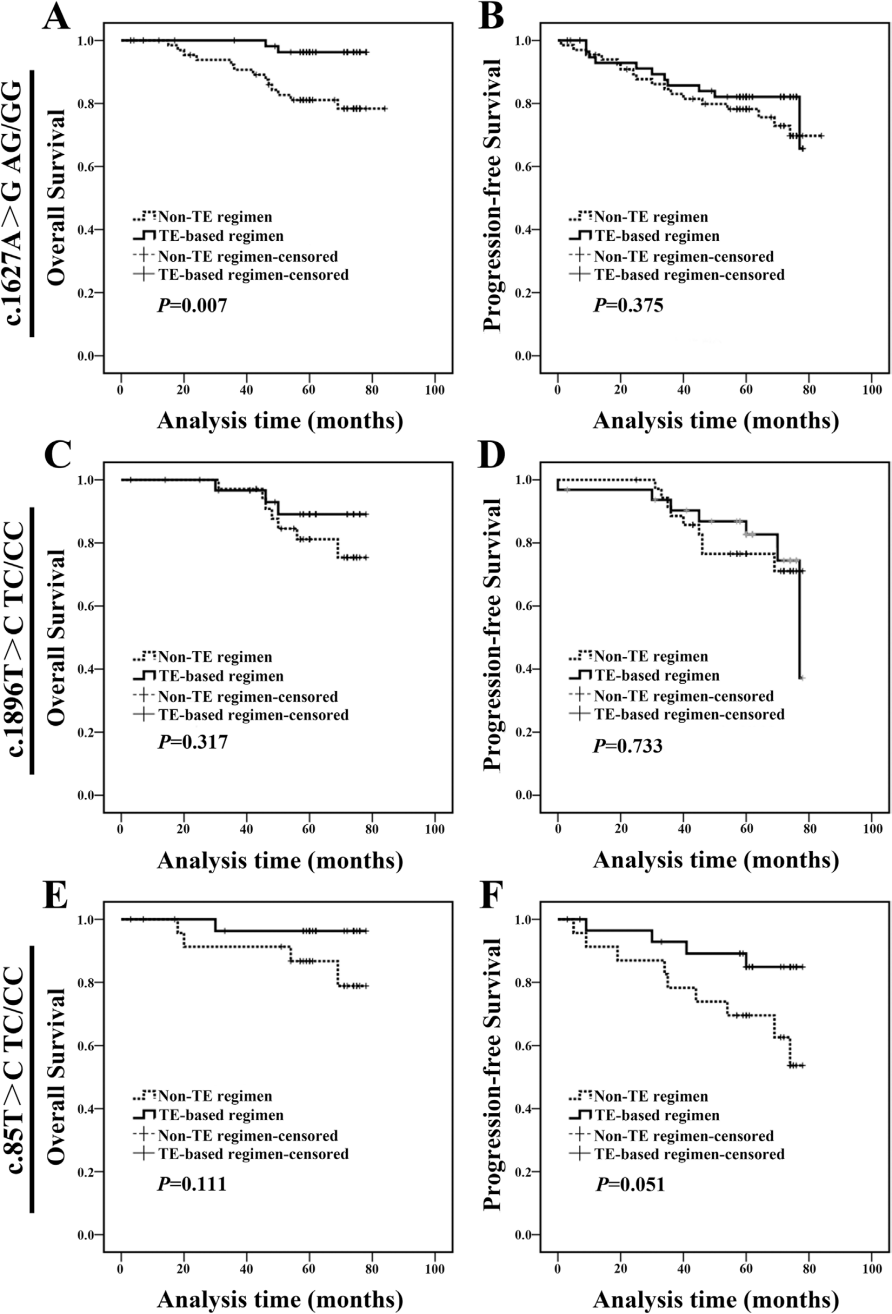
c.1627A>G AG/GG genotype breast cancer patients treated with TE-based regimen exhibited a better prognosis **(A-B)** c.1627A>G AG/GG genotype carriers treated with TE-based regimen exhibited a longer OS than those treated with non-TE regimen. **(C-D)** c.1896T>C TC/CC genotype carriers treated with TE-based regimen exhibited a similar OS and PFS with those treated with non-TE regimen. **(E-F)** c.85T>C TC/CC genotype carriers treated with TE-based regimen exhibited a similar OS and PFS compared with those treated with non-TE regimen.

Subsequently, the clinical significance of 3 polymorphisms was investigated, individually. For c.1627A>G AG/GG genotype carriers, patients treated with TE-based regimen exhibited a longer OS compared with those treated with non-TE regimen (Figure 7A and 7B). We did not find any difference in clinicopathologic characteristics between two populations at baseline (Supplementary Table 7). Meanwhile, patients treated with TE-based regimen exhibited a similar prognosis with those treated with non-TE regimen in both c.1896T>C TC/CC genotype carriers and c.85T>C TC/CC genotype carriers (Figure 7C-7F).

**Figure 8:**
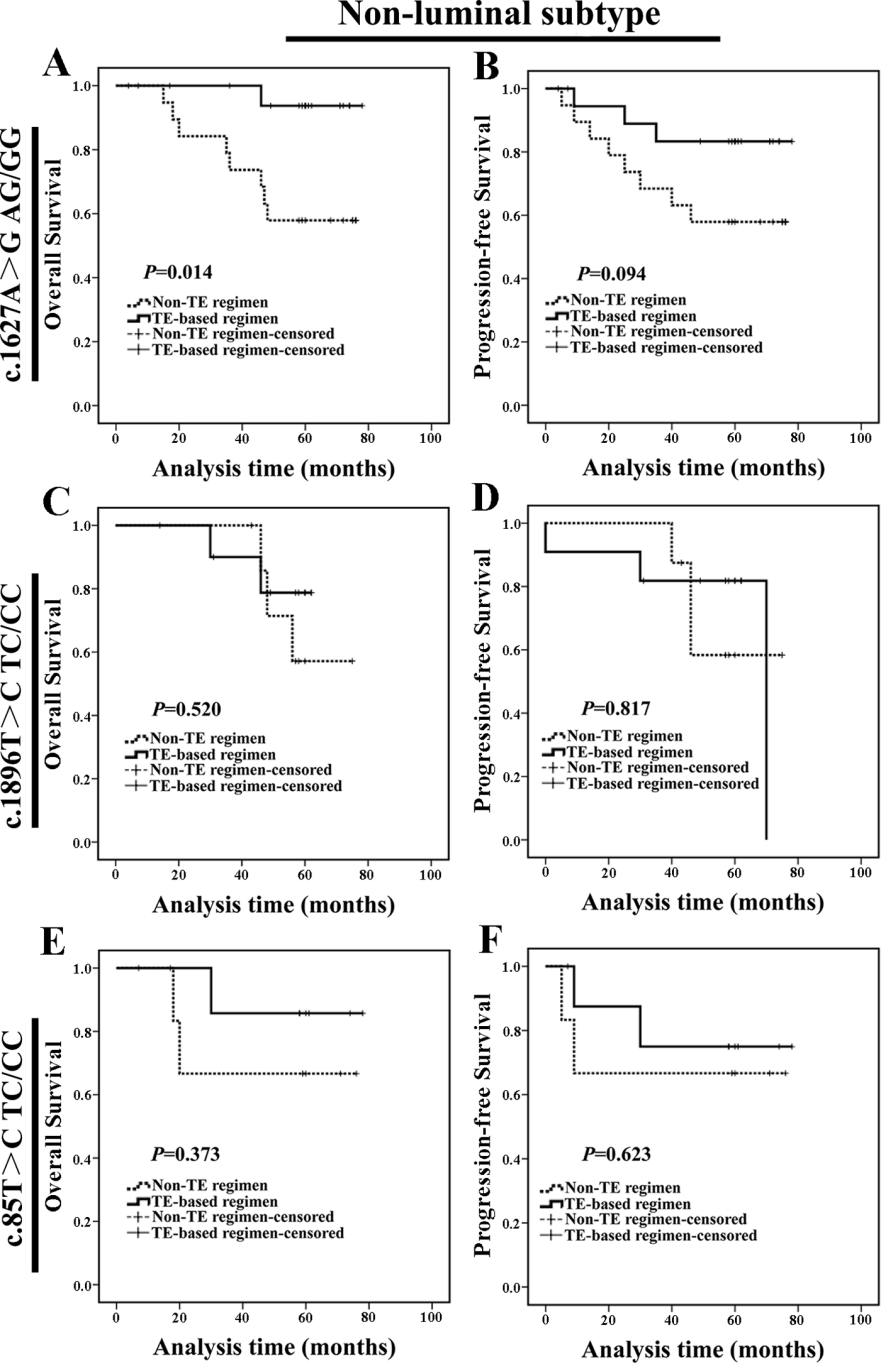
Non-luminal c.1627A>G AG/GG genotype carriers treated with TE-based regimen exhibited a better prognosis **(A-B)** For non-luminal subtype, c.1627A>G AG/GG genotype carriers treated with TE-based regimen exhibited a longer OS than those treated with non-TE regimen. **(C-D)** For non-luminal subtype, c.1896T>C TC/CC genotype carriers treated with TE-based regimen exhibited a similar OS and PFS compared with those treated with non-TE regimen. **(E-F)** For non-luminal subtype, c.85T>C TC/CC genotype carriers treated with TE-based regimen exhibited a similar OS and PFS compared with those treated with non-TE regimen.

Furthermore, for non-luminal subtype patients with c.1627A>G AG/GG genotype, those treated with TE-based regimen exhibited a better OS compared with patients treated with non-TE regimen (Figure 8A and 8B). We did not find any difference in clinicopathologic characteristics between such two populations at baseline (Supplementary Table 8). For both non-luminal c.1896T>C TC/CC and c.85T>C TC/CC genotype carriers, patients treated with TE-based regimen exhibited similar prognosis compared with those treated with non-TE regimen (Figure 8C-8F). Meanwhile, luminal carriers treated with TE-based regimen exhibited similar OS compared with those treated with non-TE regimen (Supplementary Figure 3). These results indicated that non-luminal subtype patients with c.1627A>G AG/GG could benefit from TE-based chemotherapy.

Meanwhile, Western blot analyses also were employed by using IDC tissues (8 cases carrying c.1627A>G AA genotype and 8 cases carrying c.1627A>G AG/GG genotype) and 2 primary cells (121918: c.1627A>G AA; 1028: c.1627A>G AG) derived from two non-luminal breast cancer patients (details in Table 3). Our results showed that the c.1627A>G AG/GG had no effect on protein expression (Supplementary Figure 4).

**Table 3:** Details of 2 primary breast cancer cell lines

Primary cell line	Gender	Age (years)	Histological grade	pTNM	ER status	PR status	HER2 status	Molecular subtype	*DPYD* c.1627A>G genotype
**121918**	**female**	**52**	**III**	**II**	**Negative**	**Negative**	**Negative**	**Triple negative subtype**	**AA**
**1028**	**female**	**64**	**III**	**II**	**Negative**	**Negative**	**Positive**	**HER2-overexpression subtype**	**AG**

**Figure 9:**
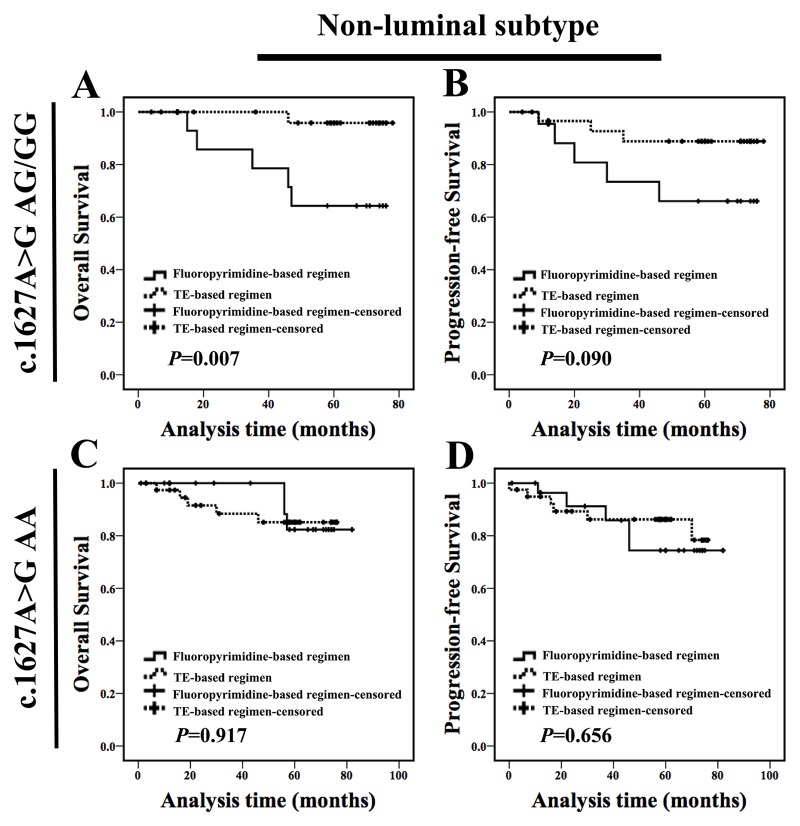
Non-luminal c.1627A>G AG/GG genotype carriers treated with fluoropyrimidines chemotherapy exhibited worse prognosis compared with those treated with TE-based regimen **(A-B)** For non-luminal subtype, c.1627A>G AG/GG genotype carriers treated with TE-based regimen exhibited a longer OS than those treated with fluoropyrimidines chemotherapy. **(C-D)** For non-luminal subtype, c.1627A>G AA genotype carriers treated with TE-based regimen exhibited a similar OS and PFS compared with those treated with fluoropyrimidines chemotherapy.

In order to confirm our above conclusion, survival analysis of another cohort of 123 non-luminal subtype patients including 54 patients with c.1627A>G AG/GG genotype and 69 patients with c.1627A>G AA genotype were performed. For those c.1627A>G AG/GG genotype carriers, patients treated with TE-based chemotherapy exhibited a better prognosis compared with those treated with fluoropyrimidines chemotherapy (OS: *P*=0.007, PFS: *P*=0.090, Figure 9A-9B), and we did not find any difference in clinicopathologic characteristics between two populations at baseline (Supplementary Table 9). Meanwhile, for those c.1627A>G AA genotype carriers, patients treated with fluoropyrimidines chemotherapy exhibited a similar survival with those treated with TE-based chemotherapy (Figure 9C-9D). All these results indicated that TE-based chemotherapy was a suitable regimen for non-luminal patients with *DPYD* c.1627A>G AG/GG genotype and fluoropyrimidine-based regimen should not be recommended for those patients.

**Figure 10:**
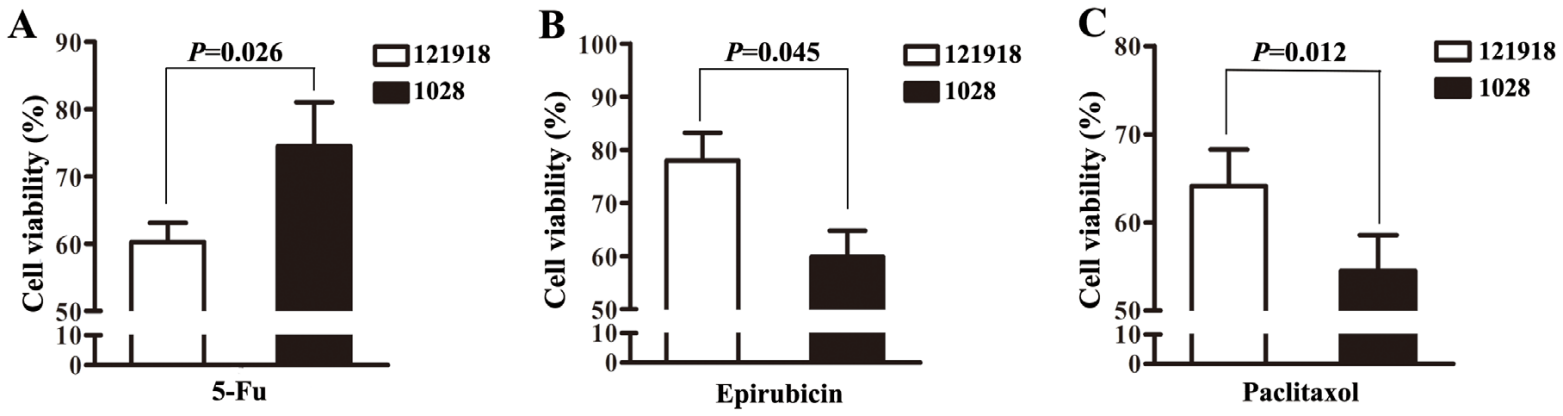
The effect of 5-Fu, epirubicin and paclitaxol on the growth of non-luminal breast cancer derived cells **(A)** After primary cells were treated with 5-Fu (1μg/ml) for 48h, cell viability of 121918 (c.1627A>G AA) and 1028 (c.1627A>G AG) was measured by MTT assay. **(B)** After primary cells were treated with epirubicin (1μg/ml) for 48h, cell viability of 121918 and 1028 was measured by MTT assay. **(C)** After primary cells were treated with paclitaxol (1μg/ml) for 48h, cell viability of 121918 and 1028 was measured by MTT assay.

Finally, both 121918 and 1028 primary cells were used for MTT assays to confirm above conclusion. After 5-Fu treatment, 1028 (c.1627A>G AG) exhibited a higher cell viability than 121918 (c.1627A>G AA) (*P*=0.026, Figure 10A). Meanwhile, 1028 exhibited a lower cell viability than 121918 with epirubicin (*P*=0.045, Figure 10B) or paclitaxel treatment (*P*=0.012, Figure 10C).

## DISCUSSION

Although more than 160 polymorphisms in *DPYD* gene have been identified to date, data derived from Chinese cancer patients is very limited [19, 20]. In the present study, we detected 5 polymorphisms including c.74A>G, c.85T>C, c.1627A>G, c.1896T>C and c.2194G>A in breast cancer patients of China north area; and the genotype frequencies of these polymorphisms in our study were similar to previous research [21, 22]. Furthermore, our research investigated the prognostic value of these polymorphisms for the first time in a large breast cancer patient population. We demonstrated for the first time that, *DPYD* gene polymorphisms could directly provide valuable prognostic information. We proposed for the first time that, TE-based chemotherapy was a suitable regimen for non-luminal breast cancer patients with *DPYD* c.1627A>G AG/GG, whereas fluoropyrimidine-based chemotherapy should not be recommended.

Actually, previous studies on *DPYD* were mainly focused on *DPYD* polymorphisms-induced severe toxicities and tried to explain this phenomenon by deregulated DPD enzyme activity [23]. Unfortunately, prior investigations did not reach agreements and some researchers even got controversial conclusion. For example, Deenen et al. failed to show any correlation between c.85T>C, c.1627A>G, c.1896T>C and fluoropyrimidine-related toxicity in colorectal cancer patients [24], whereas in other reports, a statistically higher incidence of side effects was observed in gastrointestinal malignancies with those 3 SNPs listed above [14, 25]. In this study, we found no obvious fluoropyrimidines-associated toxicity in breast cancer patients with non-wild type *DPYD*.

According to our results, non-luminal breast cancer patients carrying *DPYD* c.1627A>G AG/GG treated with fluoropyrimidine-based regimen presented a shorter OS (*P*=0.002) and PFS (*P*=0.015) (Figure 5A and 5B). These results were somewhat indirectly supported by previous study in which fluorouracil-based chemotherapy showed no effect in gastric cancer patients carrying c.1627A>G AG/GG genotype [16]. Furthermore, Gross’s research demonstrated that somatic copy number changes in *DPYD*, rather than aberrant DPD protein level, could reflect a distinct tumor profile associated with distinct outcomes in breast cancer [26].

In our retrospectively study, we were unable to assess DPD enzyme activity since it was extremely complex to collect samples of subjects. Furthermore, given the ambiguous role of c.1627A>G polymorphisms in DPD enzyme activity based on previous studies and the complexity of DPD pharmacokinetics, we suggest that screening of patients for *DPYD* c.1627A>G polymorphisms prior to administration of fluoropyrimidines will allow more appropriate and individualized approach to chemotherapy management.

The most clinically useful aspect of our results is the potential ability to identify patients with breast cancer who are insensitive to fluoropyrimidine-based regimen and likely to benefit from TE-based chemotherapy. As is well known, TE-based chemotherapy, including anthracycline and docetaxel, is widely used in breast cancer treatment. Anthracyclines are antibodies produced from the streptomyces species, which can inhibit nuclear enzyme TOPO (topoisomerase) II leading to DNA double-strand breaks and cell death [27]. Docetaxel has the ability to polymerize tubulin in the absence of GTP, which under normal conditions is an absolute requirement for microtubule polymerization. Paclitaxel-bound microtubules are unusually stable and resistant to depolymerization. The most significant cellular impact of the interference with microtubule dynamics is during the mitotic phase of the cell cycle where paclitaxel inhibits mitotic spindle dynamics leading to mitotic arrest and consequent apoptotic cell death [27]. Although the mechanism linking *DPYD* SNPs and TE-based chemotherapy response is unknown and further exploration is necessary, we speculate that accumulation of DNA damage and abnormal DNA repair might contribute to this phenomenon.

In conclusion, our study demonstrated that TE-based chemotherapy was a suitable regimen for non-luminal breast cancer patients with *DPYD* c.1627A>G AG/GG, while fluoropyrimidine-based chemotherapy should not be recommended. Our findings provided a novel strategy, which will guide clinicians to choose more precise chemotherapy treatment for breast cancer patients.

## MATERIALS AND METHODS

### Patient selection and clinical information

Paraffin-embedded specimens of 331 breast cancer patients with invasive carcinoma, diagnosed between 2010 and 2011 were reviewed and randomly selected from Department of Breast Cancer Pathology and Research Laboratory, Tianjin Medical University Cancer Institute & Hospital (Tianjin, China). The histopathology was reviewed and diagnosis of each case was confirmed independently by two pathologists according to World Health Organization (WHO) criteria. This study was approved by the Institutional Ethic Committee of Tianjin Medical University Cancer Institute & Hospital (bc2016030). All experiments were performed in accordance with relevant guidelines and regulations of Ethic Committee of Tianjin Medical University Cancer Institute & Hospital. All the patients signed informed consent for participation of the study and the use of their biological tissues.

331 patients were women aging from 23 to 89 years (mean 52.6 years) without preoperative radiation. Among them, 231 patients were luminal subtype, 51 patients were HER2-overexpression subtype and 44 were triple-negative subtype. 157 patients received TE (taxane and anthracycline) chemotherapies, 49 patients received chemotherapies with fluoropyrimidines and 70 patients received CET (cyclophosphamide, anthracycline and taxane) chemotherapy, chemotherapeutic information of 20 patients was missing, the rest (35 patients) were treated with other chemotherapy after operation. None patients presented severe toxic and side reaction of chemotherapy. A total of 320 cases were included for prognostic analyses, excluding 11 cases with no follow-up data. The follow-up was 3-84 months (Median: 58.3). Recurrences were recorded for 5 cases and 36 developed distant metastasis (bone metastasis: 13, lung metastasis: 16, liver metastasis: 9, brain metastasis: 3, kidney metastasis: 3, thyroid metastasis: 1). It was worth noting that multiple organic metastases were noted in 13 patients. 33 patients died during the follow-up period.

To further investigate our conclusion, another cohort of 123 non-luminal subtype breast cancer patients with follow-up (mean aged 52.61, range: 23-89) were selected. Among them, 52 patients received fluoropyrimidines and 71 patients received TE-based chemotherapies. Genomic DNA was isolated from tumor tissues using standard techniques and PCR-sequencing was applied to detect the SNPs status of c.1627A>G. In our survival analysis, they were divided into four groups: patients with c.1627A>G AG/GG treated with fluoropyrimidines chemotherapy (n=23); patients with c.1627A>G AG/GG treated with TE-based chemotherapy (n=31); patients with c.1627A>G AA treated with fluoropyrimidines chemotherapy (n=29) and patients with c.1627A>G AA treated with TE-based chemotherapy (n=40).

16 IDC tissues (8 cases with c.1627A>G AA and 8 cases with c.1627A>G AG/GG) were applied in the Western Blot assay. 2 primary breast cancer cells (121918 and 1028, derived from 2 non-luminal breast cancer patients, respectively) were applied in the Western Blot assay and MTT assay. 121918 cells was *DPYD* c.1627A>G AA and 1028 cells was *DPYD* c.1627A>G AG (details in Table 3). All patients were women without preoperative chemotherapy or radiation.

### The pathological diagnosis criteria of breast cancer molecular subtype

Molecular subtype classification of breast cancer according to the St Gallen recommendation was based on the immunohistochemistry analysis of ER, PR and HER2 [28]. Luminal subtype (ER+ or/and PR+, HER2- or HER2+), non-luminal subtype (ER- and PR-, HER2- or HER2+). We provided representative immunohistochemical images of luminal and non-luminal subtype in Supplementary Figure 5.

### Determination of 5 *DPYD* SNPs status

5 *DPYD* single nucleotide polymorphisms (SNPs) status (c.74A>G, c.85T>C, c.1627A>G, c.1896T>C and c.2194G>A) were determined using the polymerase chain reaction (PCR)-sequencing method. The c.74A>G is a transition from A to G at nucleotide position 74, resulting to a Histidine change to an Arginine. The c.85T>C (rs1801265) is a transition from T to C at nucleotide position 85 with an amino acid change from Cysteine to Argnine. The c.1627A>G (rs1801159) is a transition from A to G at nucleotide position 1627, leading to an Isoleucine change to a Valine. The c.1896T>C (rs17376848) is a synonymous SNP, which has a transition from T to C at nucleotide position 1896. The c.2194G>A (rs1801160) is a transition from G to A at nucleotide position 2194, leading to a Valine to an Isoleucine.

Genomic DNA was isolated from tumor tissues of invasive breast carcinoma patients using standard techniques. PCR-sequencing was applied to detect 5 SNPs status. In brief, 4 coding regions of *DPYD* (NM_000110.3) were amplified by using 4 polymerase chain reaction (PCR) mixtures respectively. *DPYD* c.74A>G and c.85T>C were amplified in the same reaction, so 5 SNPs were amplified by 4 reaction mixtures. For the reaction of c.74A>G and c.85T>C, PCR program was used as follows: initial denaturation (94°C, 2 min); followed by 30 PCR cycles (98°C, 20s; 70°C, 30s; 70°C, 40s) and a final extension (72°C, 10 min). For the reaction of c.1627A>G, PCR program was used as follows: initial denaturation (94°C, 2 min); followed by 30 PCR cycles (98°C, 20s; 52°C, 30s; 70°C, 40s) and a final extension (72°C, 10 min). For the reaction of c.1896T>C, PCR program was used as follows: initial denaturation (94°C, 2 min); followed by 30 PCR cycles (98°C, 20s; 59.1°C, 30s; 70°C, 40s) and a final extension (72°C, 10 min). For the reaction of c.2194G>A, PCR program was used as follows: initial denaturation (94°C, 2 min); followed by 30 PCR cycles (98°C, 20s; 56.1°C, 30s; 70°C, 40s) and a final extension (72°C, 10 min). Reactions were carried out in a total volume of 25 μl, using 100 ng of genomic DNA as templates. After amplification, PCR products were sequenced by company (GENEWIZ, Suzhou, China). Primers for amplifications were all designed and synthesized by GENEWIZ, and primer sequences are presented in Supplementary Table 1.

### Statistical methods

The SPSS 13.0 software package (SPSS, Chicago, IL, USA) was used for statistical analysis. The Chi-square (χ^2^) test or Fisher’s exact test was used for analysis of Hardy-Weinberg equilibrium and correlations between two variables were evaluated by Spearman rank correlation test. Progression-free survival (PFS) was defined as the time from surgery to recurrence or cancer-specific death, whichever occurred first. Overall survival (OS) was calculated from pathological diagnosis to the date of last contact or death from breast carcinoma. Survival analysis was performed according to the Kaplan-Meier method and assessed using the log-rank test. All statistical tests were 2-tailed and *P*< 0.05 was regarded as significant.

### Western blot

Tissues or cells were lysed in SDS lysis buffer on ice. Equal amounts of cell lysates were loaded and separated by SDS-PAGE, and proteins were transferred onto nitrocellulose membranes and incubated with the primary DPD antibody (1:1000, ab54797, abcam, USA) overnight at 4°C. Membranes were then treated with secondary antibodies. Blots were analyzed by Licor Odyssey infrared imaging.

### MTT assay

3×10^4^ cells were seeded in 24-well plates. After overnight culture to allow cells to adhere, culture medium containing 5-Fu, paclitaxel or epirubicin at indicated concentration was added for an additional 48 h. Cells were then incubated with 500 μl of the MTT stock solution (5 mg/ml) for 4 hours. Finally, medium was removed and the converted dye was solubilized with dimethyl sulfoxide. The absorbance of the converted dye was measured at a wave length of 570 nm. The absorbance in the untreated control group was regarded as 100% cell viability.

### Author contributions

Conception and design: Yongjie Ma, Feng Gu and Li Fu. Acquisition of data: Yong Huang, Huikun Zhang and Fengxia Qin. Performed the experiments: Yong Huang, Huikun Zhang and Fengxia Qin. Analysis of data: Yong Huang. Statistical analyses: Feng Yu and Limin Yang. Writing of manuscript: Fengxia Qin and Yongjie Ma. Preparation of tables and figures: Fengxia Qin and Xiaoli Liu. All authors reviewed the manuscript.

## SUPPLEMENTARY MATERIALS FIGURES AND TABLES


