# Supplementary material for: Effect of dihydropyrimidine dehydrogenase single nucleotide polymorphisms on prognosis of breast cancer patients with chemotherapy
Source: Oncotarget. 2017 Dec 8;8(67):112060–75. doi: 10.18632/oncotarget.23033 (PMC5762379; doi:10.18632/oncotarget.23033)
Supplement: Supplementary file 1 [file oncotarget-08-112060-s001.pdf]

Effect of dihydropyrimidine dehydrogenase single nucleotide polymorphisms on prognosis of breast cancer patients with chemotherapy

SUPPLEMENTARY MATERIALS

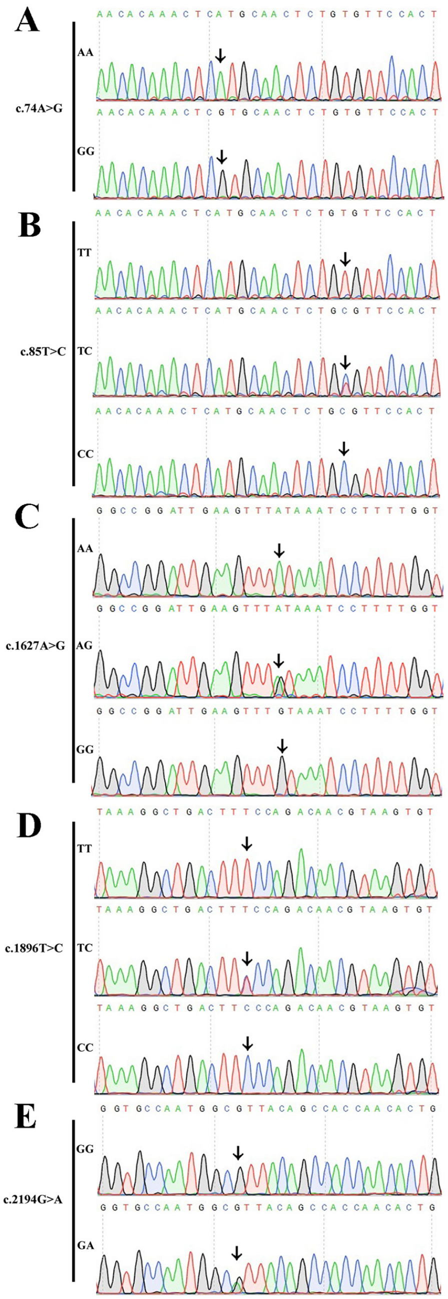

Supplementary Figure 1: Genomic DNA sequencing of the 5 SNPs of *DPYD*. **(A)** c.74A>G. **(B)** c.85T>C. **(C)** c.1627A>G. **(D)** c.1896T>C. **(E)** c.2194G>A.

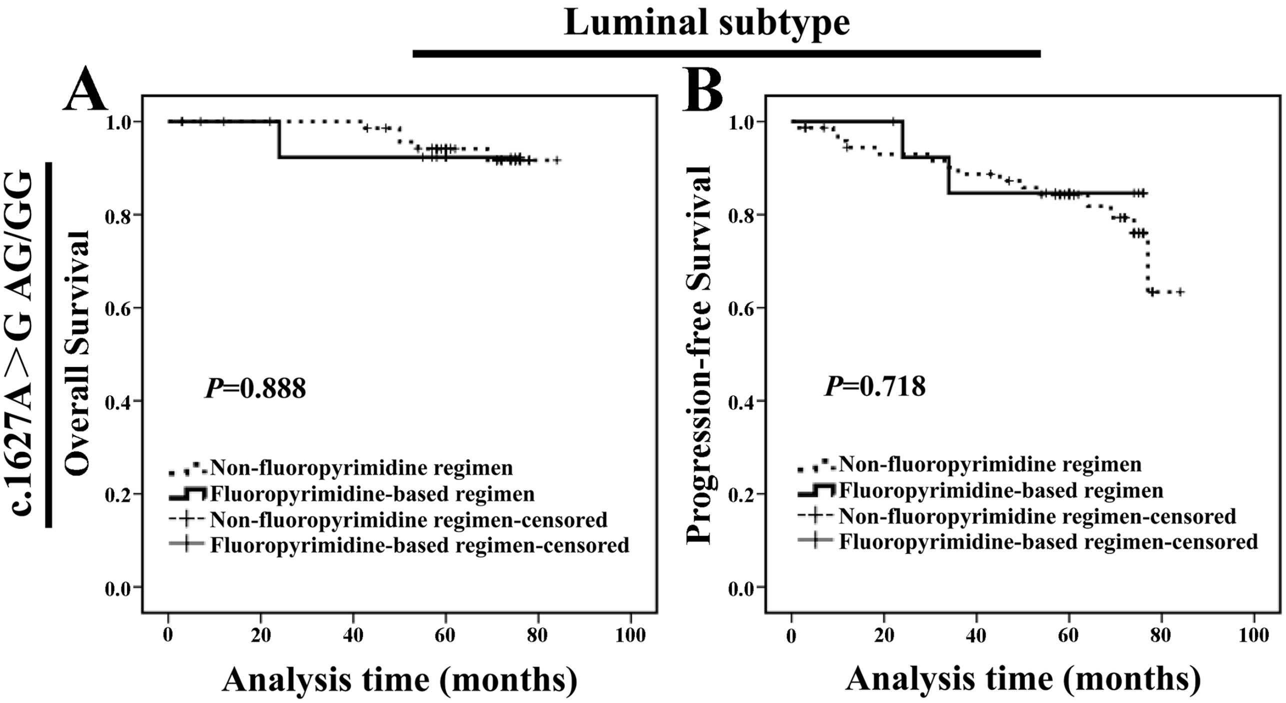

Supplementary Figure 2: Survival analysis of luminal breast cancer patients treated with fluoropyrimidine-based regimen with c.1627A>G AG/GG genotype. **(A)** For luminal subtype, c.1627A>G AG/GG genotype carriers treated with fluoropyrimidine-based regimen exhibited a similar OS compared with those treated with non-fluoropyrimidine regimen. **(B)** For luminal subtype, c.1627A>G AG/GG genotype carriers treated with fluoropyrimidine-based regimen exhibited a similar PFS compared with those treated with non-fluoropyrimidine regimen.

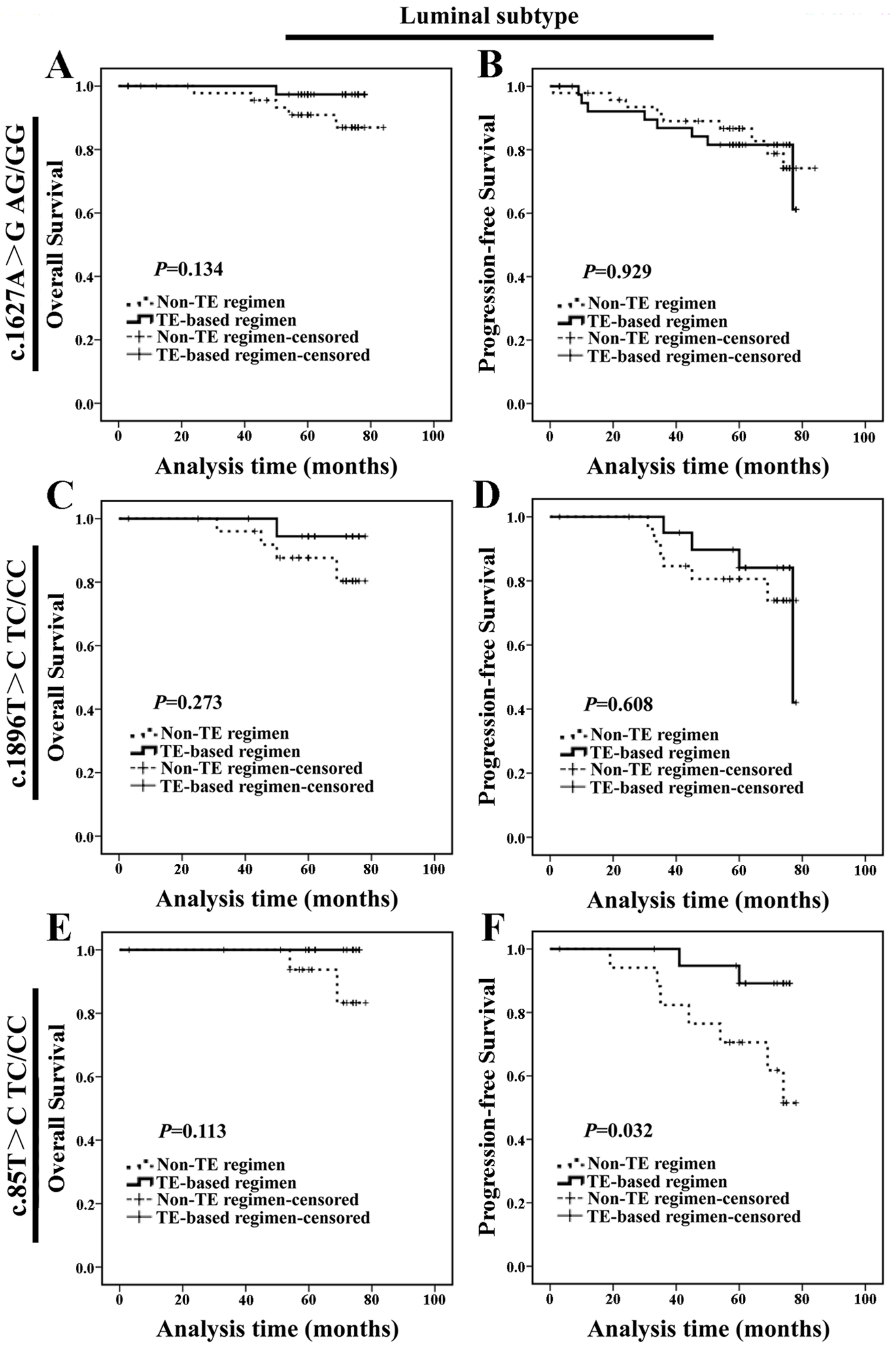

Supplementary Figure 3: Survival analysis of luminal breast cancer patients treated with TE-based regimen with 3 different *DPYD* SNPs. (A-B) For luminal subtype, c.1627A>G AG/GG genotype carriers treated with TE-based regimen exhibited a similar OS and PFS compared with those treated with non-TE regimen. **(C-D)** For luminal subtype, c.1896T>C TC/CC genotype carriers treated with TE-based regimen exhibited a similar OS and PFS compared with those treated with non-TE regimen. **(E-F)** For luminal subtype, c.85T>C TC/CC genotype carriers treated with TE-based regimen exhibited a similar OS compared with those treated with non-TE regimen.

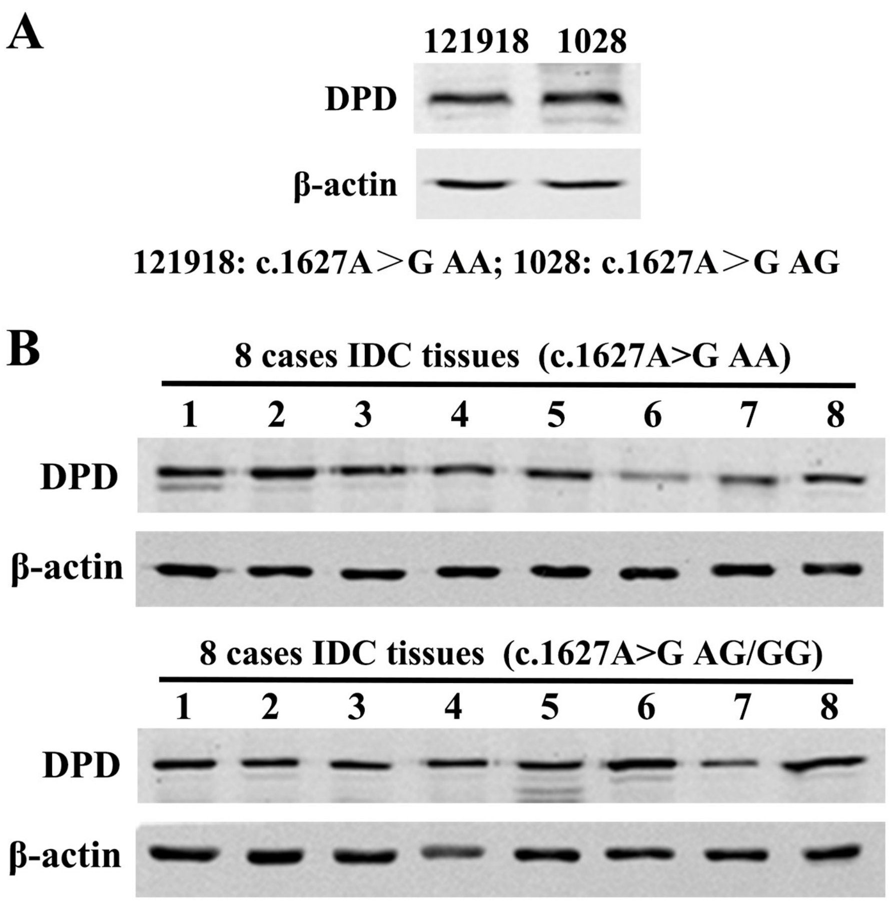

Supplementary Figure 4: The effect of c.1627A>G AG/GG on the expression of DPD protein. (A) Western blot analysis of DPD expression in non-luminal breast cancer derived cells (121918: c.1627A>G AA; 1028: c.1627A>G AG). **(B)** Western blot analysis of DPD expression in breast cancer tissues from IDC patients with c.1627A>G AA genotype (8 cases) and c.1627A>G AG/GG genotype (8 cases), respectively. β-actin was used as a loading control.

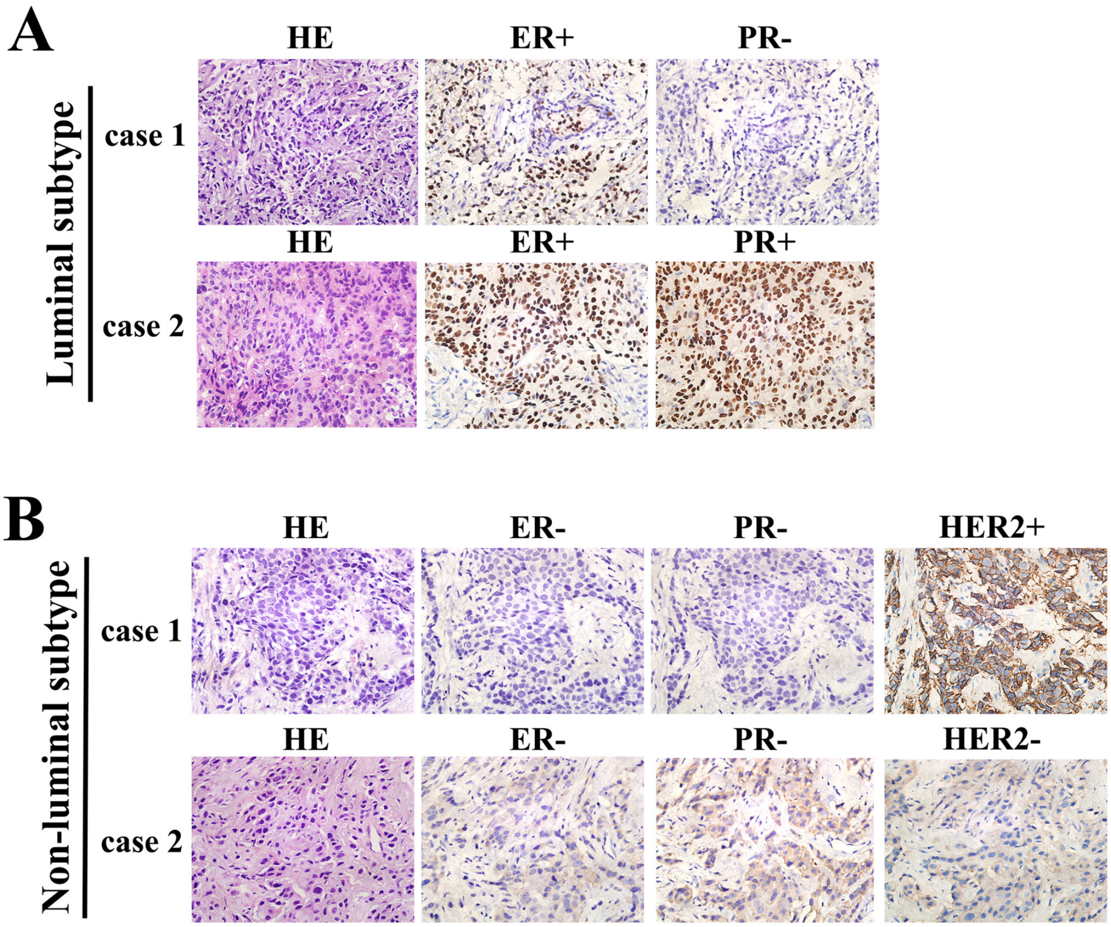

Supplementary Figure 5: Representative immunohistochemical images of luminal and non-luminal breast cancer patients. **(A)** Representative immunohistochemical images of luminal breast cancer patients (2 cases, magnification 400×). **(B)** Representative immunohistochemical images of non-luminal breast cancer patients (2 cases, magnification 400×).

Supplementary Table 1: The association between *DPYD* SNPs status and clinicopathological characteristics

Pathological features	Cases	*DPYD* gene		r_s	*P* value
		Wild type	Non-wild type		
Age	331			-0.054	0.323
<50		44 (33.3)	88 (66.7)		
≥50		77 (38.7)	122 (61.3)		
Histological grade[a]	288			-0.020	0.737
GradeI		5 (41.7)	7 (58.3)		
Grade II		72 (33.8)	141 (66.2)		
Grade III		24 (38.1)	39 (61.9)		
Tumor size[a]	306			-0.087	0.129
≤5		93 (34.7)	175 (65.3)		
>5		18 (47.4)	20 (52.6)		
Lymph node status[a]	306			-0.030	0.598
pN0		55 (37.7)	91 (62.3)		
pN1		17 (22.7)	58 (77.3)		
pN2		23 (48.9)	24 (51.1)		
pN3		16(42.1)	22 (57.9)		
ER[a]	329			0.033	0.550
Negative		40 (38.8)	63 (61.2)		
Positive		80 (35.4)	146 (64.6)		
PR[a]	329			0.067	0.225
Negative		50 (40.7)	73 (59.3)		
Positive		70 (34.0)	136 (66.0)		
HER2[a]	327			0.014	0.798
Negative		79 (36.6)	137 (63.4)		
Positive		39 (35.1)	72 (64.9)		
Molecular subtype[a]	326			0.023	0.683
Luminal		82 (35.5)	149 (64.5)		
Non-luminal		36 (37.9)	59 (62.1)		
Recurrence or distant metastasis[a]	324			0.095	0.087
Negative		108 (38.2)	175 (61.8)		
Positive		10 (24.4)	31 (75.6)		

Wild type: patients with c.85T>C TT, c.1627A>G AA and c.1896T>C TT simultaneously.
Non-wild type: patients with whichever of mutations at c.85T>C, c.1627A>G or c.1896T>C.
[a]Some missing data.
P values were calculated by Spearman's Rank-Correlation test.

Supplementary Table 2: Baseline characteristics of the breast cancer patients with non-wild type *DPYD*

Pathological features	Cases	Chemotherapy		χ^2	*P* value
		Non-Fluoropyrimidine regimen	Fluoropyrimidine -based regimen		
Age	197			0.638	0.424[b]
<50		72 (85.7)	12 (14.3)		
≥50		92 (81.4)	21 (18.6)		
Histological grade[a]	175			2.373	0.305[b]
Grade I		5 (71.4)	2 (28.6)		
Grade II		111 (85.4)	19 (14.6)		
Grade III		29 (76.3)	9 (23.7)		
Tumor size[a]	183				0.191[c]
≤5		140 (85.4)	24 (14.6)		
>5		14 (73.7)	5 (26.3)		
Lymph node status[a]	183				0.958[c]
pN0		73 (83.0)	15 (17.0)		
pN1		44 (86.3)	7 (13.7)		
pN2		19 (82.6)	4 (17.4)		
pN3		18 (85.7)	3 (14.3)		
ER status	197			1.495	0.221[b]
Negative		47 (78.3)	13 (21.7)		
Positive		117 (85.4)	20 (14.6)		
PR status	197			0.822	0.365[b]
Negative		56 (80.0)	14 (20.0)		
Positive		108 (85.0)	19 (15.0)		
HER2 status	197			2.663	0.103[b]
Negative		109 (86.5)	17 (13.5)		
Positive		55 (77.5)	16 (22.5)		

[a]Some missing data.
[b]Two-sided χ^2 test.
[c]Fisher's exact test.

Supplementary Table 3: Baseline characteristics of the non-luminal subtype patients with non-wild type *DPYD*

Pathological features	Cases	Chemotherapy		*P* value[b]
		Non-Fluoropyrimidine regimen	Fluoropyrimidine -based regimen	
Age	56			0.258
<50		13 (92.9)	1 (7.1)	
≥50		31 (73.8)	11 (26.2)	
Histological grade[a]	50			0.506
Grade II		24 (80.0)	6 (20.0)	
Grade III		14 (70.0)	6 (30.0)	
Tumor size[a]	52			0.120
≤5		38 (84.4)	7 (15.6)	
>5		4 (57.1)	3 (42.9)	
Lymph node status[a]	52			0.419
pN0		26 (86.7)	4 (13.3)	
pN1		6 (75.0)	2 (25.0)	
pN2		5 (62.5)	3 (37.5)	
pN3		5 (83.3)	1 (16.7)	
HER2 status	56			0.322
Negative		20 (87.0)	3 (13.0)	
Positive		24 (72.7)	9 (27.3)	

[a]Some missing data.
[b]Fisher's exact test.

Supplementary Table 4: Baseline characteristics of breast cancer patients with c.1627A>G AG/GG genotype

Pathological features	Cases	Chemotherapy		χ^2	*P* value
		Non-Fluoropyrimidine regimen	Fluoropyrimidine -based regimen		
Age	131			0.865	0.352[b]
<50		49 (86.0)	8 (14.0)		
≥50		59 (79.7)	15 (20.3)		
Histological grade[a]	117				0.115[c]
Grade I		3 (60.0)	2 (40.0)		
Grade II		73 (85.9)	12 (14.1)		
Grade III		20 (74.1)	7 (25.9)		
Tumor size[a]	125				0.059[c]
≤5		95 (85.6)	16 (14.4)		
>5		9 (64.3)	5 (35.7)		
Lymph node status[a]	125				0.919[c]
pN0		44 (80.0)	11 (20.0)		
pN1		35 (85.4)	6 (14.6)		
pN2		12 (85.7)	2 (14.3)		
pN3		13 (86.7)	2 (13.3)		
ER status	131			0.503	0.478[b]
Negative		34 (79.1)	9 (20.9)		
Positive		74 (84.1)	14 (15.9)		
PR status	131			0.074	0.785[b]
Negative		39 (81.2)	9 (18.8)		
Positive		69 (83.1)	14 (16.9)		
HER2 status	131			0.562	0.454[b]
Negative		70 (84.3)	13 (15.7)		
Positive		38 (79.2)	10 (20.8)		

[a]Some missing data.
[b]Two-sided χ^2 test.
[c]Fisher's exact test.

Supplementary Table 5: Baseline characteristics of the non-luminal subtype patients with c.1627A>G AG/GG genotype

Pathological features	Cases	Chemotherapy		*P* value[b]
		Non-Fluoropyrimidine regimen	Fluoropyrimidine -based regimen	
Age	40			0.404
<50		9 (90.0)	1 (10.0)	
≥50		22 (73.3)	8 (26.7)	
Histological grade[a]	36			0.443
Grade II		17 (81.0)	4 (19.0)	
Grade III		10 (66.7)	5 (33.3)	
Tumor size[a]	37			0.068
≤5		27 (87.1)	4 (12.9)	
>5		3 (50.0)	3 (50.0)	
Lymph node status[a]	37			0.485
pN0		16 (88.9)	2 (11.1)	
pN1		6 (75.0)	2 (25.0)	
pN2		3 (60.0)	2 (40.0)	
pN3		5 (83.3)	1 (16.7)	
HER2 status	40			0.453
Negative		12 (85.7)	2 (14.3)	
Positive		19 (73.1)	7 (26.9)	

[a]Some missing data.
[b]Fisher's exact test.

Supplementary Table 6: Baseline characteristics of the breast cancer patients with non-wild type *DPYD*

Pathological features	Cases	Chemotherapy		χ^2	*P* value
		Non-TE regimen	TE-based regimen		
Age	197			0.463	0.496[b]
<50		39 (46.4)	45 (53.6)		
≥50		58 (51.3)	55 (48.7)		
Histological grade[a]	175				0.568[c]
Grade I		4 (57.1)	3 (42.9)		
Grade II		60 (46.2)	70 (53.8)		
Grade III		21(55.3)	17 (44.7)		
Tumor size[a]	183			0.101	0.751[b]
≤5		80 (48.8)	84 (51.2)		
>5		10 (52.6)	9 (47.4)		
Lymph node status[a]	183			2.430	0.488[b]
pN0		42 (47.7)	46 (52.3)		
pN1		26 (51.0)	25 (49.0)		
pN2		9 (39.1)	14 (60.9)		
pN3		13 (61.9)	8 (38.1)		
ER status	197			0.620	0.431[b]
Negative		27 (45.0)	33 (55.0)		
Positive		70 (51.1)	67 (48.9)		
PR status	197			0.540	0.463[b]
Negative		32 (45.7)	38 (54.3)		
Positive		65 (51.2)	62 (48.8)		
HER2 status	197			0.367	0.545[b]
Negative		60 (47.6)	66 (52.4)		
Positive		37 (52.1)	34 (47.9)		

[a]Some missing data.
[b]Two-sided χ^2 test.
[c]Fisher's exact test.

Supplementary Table 7: Baseline characteristics of breast cancer patients with c.1627A>G AG/GG genotype

Pathological features	Cases	Chemotherapy		χ^2	*P* value
		Non-TE regimen	TE-based regimen		
Age	131			0.130	0.718[b]
<50		29 (50.9)	28 (49.1)		
≥50		40 (54.1)	34 (45.9)		
Histological grade[a]	117				0.352[c]
Grade I		4 (80.0)	1 (20.0)		
Grade II		42 (49.4)	43 (50.6)		
Grade III		16 (59.3)	11 (40.7)		
Tumor size[a]	125			0.050	0.824[c]
≤5		59 (53.2)	52 (46.8)		
>5		7 (50.0)	7 (50.0)		
Lymph node status[a]	125			1.125	0.771[b]
pN0		28 (50.9)	27 (49.1)		
pN1		23 (56.1)	18 (43.9)		
pN2		6 (42.9)	8 (57.1)		
pN3		9 (60.0)	6 (40.0)		
ER status	131			0.378	0.539[b]
Negative		21 (48.8)	22 (51.2)		
Positive		48 (54.5)	40 (45.5)		
PR status	131			0.011	0.918[b]
Negative		25 (52.1)	23 (47.9)		
Positive		44 (53.0)	39 (47.0)		
HER2 status	131			0.217	0.641[b]
Negative		45 (54.2)	38 (45.8)		
Positive		24 (50.0)	24 (50.0)		

[a]Some missing data.
[b]Two-sided χ^2 test.
[c]Fisher's exact test.

Supplementary Table 8: Baseline characteristics of the non-luminal subtype patients (n=40) with c.1627A>G AG/GG genotype

Pathological features	Cases	Chemotherapy		χ^2	*P* value
		Non-TE regimen	TE-based regimen		
Age	40			0.533	0.465[b]
<50		6 (60.0)	4 (40.0)		
≥50		14 (46.7)	16 (53.3)		
Histological grade[a]	36			0.003	0.955[b]
Grade II		11 (52.4)	10 (47.6)		
Grade III		8 (53.3)	7 (46.7)		
Tumor size[a]	37				0.383[c]
≤5		13 (41.9)	18 (58.1)		
>5		4 (66.7)	2 (33.3)		
Lymph node status[a]	37				0.669[c]
pN0		7 (38.9)	11 (61.1)		
pN1		4 (50.0)	4 (50.0)		
pN2		2 (40.0)	3 (60.0)		
pN3		4 (66.7)	2 (33.3)		
HER2 status	40			0.440	0.507[b]
Negative		6 (42.9)	8 (57.1)		
Positive		14 (53.8)	12 (46.2)		

[a]Some missing data.
[b]Fisher's exact test.
[c]Fisher's exact test.

Supplementary Table 9: Baseline characteristics of the non-luminal subtype patients (n=54) with c.1627A>G AG/GG genotype

Pathological features	Cases	Chemotherapy		χ^2	*P* value
		Fluoropyrimidine -based regimen	TE -based regimen		
Age	54			0.980	0.322[b]
<50		8 (53.3)	7 (46.7)		
≥50		15 (38.5)	24 (61.5)		
Histological grade[a]	51[a]			2.951	0.086[b]
Grade II		11 (35.5)	20 (64.5)		
Grade III		12 (60.0)	8 (40.0)		
Tumor size[a]	51[a]				0.214[c]
≤5		17 (37.8)	28 (62.2)		
>5		4 (66.7)	2 (33.3)		
Lymph node status[a]	51[a]			0.907	0.341[b]
negative		14 (46.7)	16 (53.3)		
positive		7 (33.3)	14 (66.7)		

[a]Some missing data.
[b]Two-sided χ^2 test.
[c]Fisher's exact test.
